# Supplementary material for: Chronic Glaucoma Using Biodegradable Microspheres to Induce Intraocular Pressure Elevation. Six-Month Follow-Up
Source: Biomedicines. 2021 Jun 16;9(6):682. doi: 10.3390/biomedicines9060682 (PMC8235213; doi:10.3390/biomedicines9060682)
Supplement: Supplementary file 1 [file biomedicines-09-00682-s001.zip › biomedicines-1239124-supplementary.pdf]

biomedicines

MDPI

Supplementary Materials

Chronic Glaucoma Using Biodegradable Microspheres to Induce Intraocular Pressure Elevation. Six-Month Follow-Up

Table S1. Structural analysis of neuroretina by optical coherence tomography, evaluating the left eyes of animals that had previously received the Ms20/10 model in the right eye.

		LEFT EYE, MICROSPHERE 20/10 MODEL											
**OCT PARAMETERS**	**BASELINE**	**8 w**			**12 w**			**18 w**			**24 w**		
**(μm)**	**Mean ± SD**	**Mean ± SD**	**%Ch**	***p***	**Mean±SD**	**%Ch**	***p***	**Mean ± SD**	**%Ch**	***p***	**Mean ± SD**	**%Ch**	***p***
**RETINAL THICKNESS**													
CENTRAL	275.13 ± 13.09	251.50 ± 7.76	−8.59	0.080	267.20±16.23	−2.88	0.416	265.40 ± 11.14	−3.54	**0.039**	263.00 ± 11.33	−4.41	0.173
INNER INFERIOR	255.71 ± 7.21	239.00 ± 5.29	−6.53	**0.012**[#]	249.60±6.10	−2.39	**0.042**	239.80 ± 3.70	−6.22	**0.043**	243.33 ± 9.83	−4.84	0.075
OUTER INFERIOR	245.67 ± 4.77	231.50 ± 4.50	−5.77	**0.005**[#]	239.20±6.38	−2.63	0.279	228.00 ± 3.67	−7.19	**0.042**	236.50 ± 6.18	−3.73	**0.028**
INNER SUPERIOR	251.50 ± 5.87	235.00 ± 9.66	−6.56	**0.040**	241.60±10.33	−3.94	0.138	236.80 ± 8.95	−5.84	**0.042**	247.50 ± 9.33	−1.59	0.115
OUTER SUPERIOR	251.29 ± 6.44	241.00 ± 9.76	−4.09	0.058	245.00±5.14	−2.50	0.588	240.00 ± 7.34	−4.49	0.068	253.50 ± 7.63	0.88	0.600
INNER NASAL	254.75 ± 8.71	244.00 ± 10.86	−4.22	0.228	255.60±10.35	0.33	0.686	244.00 ± 9.97	−4.22	**0.043**	252.33 ± 13.56	−0.95	0.599
OUTER NASAL	246.79 ± 5.31	241.00 ± 13.95	−2.35	0.387	246.00±7.96	−0.32	0.786	233.80 ± 2.86	−5.26	**0.042**	250.00 ± 14.95	1.30	0.416
INNER TEMPORAL	250.25 ± 7.57	232.75 ± 6.99	−6.99	**0.027**	242.60±7.02	−3.06	0.080	236.60 ± 5.32	−5.45	**0.042**	242.33 ± 10.53	−3.16	0.104
OUTER TEMPORAL	247.88 ± 5.61	236.50 ± 6.40	−4.59	**0.028**	244.80±6.05	−1.24	0.786	236.20 ± 3.11	−4.71	**0.043**	247.50 ± 9.58	−0.15	0.686
TOTAL VOLUME	1.71 ± 0.36	1.68 ± 0.17	−1.75	0.134	1.75±0.41	2.34	**0.042**	1.69 ± 0.14	−1.17	0.496	1.71 ± 0.13	−0.29	**0.027**
**RNFL THICKNESS**													
GLOBAL	46.00 ± 4.51	39.25 ± 2.98	−14.67	0.059	45.00 ± 4.79	−2.17	0.225	38.40 ± 1.81	−16.52	**0.043**	42.50 ± 7.47	−7.61	0.400
INFERIOR TEMPORAL	43.21 ± 7.53	37.00 ± 3.16	−14.37	**0.022**	44.40 ± 6.10	2.75	0.686	35.00 ± 4.30	−19.00	**0.043**	43.17 ± 11.97	−0.09	0.752
INFERIOR NASAL	46.83 ± 6.37	42.50 ± 5.32	−9.25	0.252	49.80 ± 11.45	6.34	0.893	38.40 ± 5.17	−18.00	**0.042**	53.17 ± 17.10	13.54	0.753
SUPERIOR TEMPORAL	50.38 ± 8.94	37.50 ± 7.72	−25.57	**0.024**	49.40 ± 5.94	−1.95	0.144	42.40 ± 4.98	−15.84	**0.034**	39.00 ± 3.28	−22.59	**0.027**
SUPERIOR NASAL	41.13 ± 7.18	34.50 ± 10.63	−16.12	0.188	37.40 ± 8.64	−9.07	0.345	33.40 ± 6.42	−18.79	**0.042**	36.00 ± 10.93	−12.47	0.093
NASAL	43.67 ± 5.91	43.00 ± 5.35	−1.53	0.287	40.60 ± 9.31	−7.03	0.715	40.00 ± 3.87	−8.40	0.141	43.50 ± 17.01	−0.39	0.752

TEMPORAL	49.54 ± 8.37	37.50 ± 4.20	−24.30	**0.033**	49.60 ± 9.86	0.12	0.080	40.20 ± 3.03	−18.85	**0.042**	41.33 ± 7.14	−16.57	**0.027**
**GCL THICKNESS**													
CENTRAL	23.46 ± 2.04	17.75 ± 3.30	−24.34	0.058	17.40 ± 1.81	−25.83	**0.043**	19.60 ± 2.70	−16.45	**0.042**	19.00 ± 2.68	−19.01	**0.042**
INNER INFERIOR	27.83 ± 1.52	23.00 ± 2.70	−17.36	0.115	25.80 ± 1.48	−7.29	0.066	24.60 ± 1.34	−11.61	**0.034**	24.00 ± 1.67	−13.76	**0.027**
OUTER INFERIOR	27.00 ± 1.21	22.25 ± 3.20	−17.59	0.112	24.20 ± 2.58	−10.37	0.059	24.20 ± 0.83	−10.37	**0.039**	23.00 ± 2.19	−14.81	**0.027**
INNER SUPERIOR	27.04 ± 1.42	18.75 ± 4.78	−30.66	**0.049**	22.20 ± 1.92	−17.90	**0.042**	22.00 ± 0.70	−18.64	**0.042**	21.67 ± 2.16	−19.86	**0.027**
OUTER SUPERIOR	25.96 ± 1.70	20.25 ± 1.50	−22.00	**0.028**	24.00 ± 3.00	−7.55	0.336	24.40 ± 1.81	−6.01	0.078	23.33 ± 2.94	−10.13	0.078
INNER NASAL	26.54 ± 1.91	23.75 ± 0.95	−10.51	0.135	22.20 ± 2.28	−16.35	**0.042**	23.00 ± 1.87	−13.34	**0.042**	21.67 ± 1.96	−18.35	**0.027**
OUTER NASAL	26.79 ± 1.50	23.50 ± 2.38	−12.28	**0.005** #	23.00 ± 2.82	−14.15	0.102	24.00 ± 1.00	−10.41	**0.039**	21.20 ± 1.30	−20.87	**0.042**
INNER TEMPORAL	26.88 ± 1.65	20.50 ± 7.04	−23.71	0.239	23.20 ± 2.49	−13.66	**0.043**	24.20 ± 1.64	−9.94	**0.041**	23.33 ± 1.21	−13.17	**0.024**
OUTER TEMPORAL	27.38 ± 1.31	22.00 ± 6.78	−19.65	0.273	25.40 ± 1.34	−7.23	0.063	25.00 ± 0.70	−8.69	**0.038**	24.17 ± 0.98	−11.72	**0.026**
TOTAL VOLUME	23.46 ± 2.04	17.75 ± 3.30	−24.34	0.058	17.40 ± 1.81	−25.83	**0.043**	19.60 ± 2.70	−16.45	**0.042**	19.00 ± 2.68	−19.01	**0.042**

Abbreviations: OCT: optical coherence tomography; RNFL: retinal nerve fiber layer; GCL: ganglion cell layer; thickness in microns (μm); mean ±S D (SD: standard deviation); %Ch: percentage change in thickness loss (with respect to baseline); $p < 0.05$ statistical significance (in bold); $p < 0.02$ # statistical significance with Bonferroni correction for multiple comparisons
